# Poor oral hygiene and dental caries predict high mortality rate in hemodialysis: a 3-year cohort study

**DOI:** 10.1038/s41598-020-78724-1

**Published:** 2020-12-14

**Authors:** Koji Mizutani, Risako Mikami, Tomohito Gohda, Hiromichi Gotoh, Norio Aoyama, Takanori Matsuura, Daisuke Kido, Kohei Takeda, Yuichi Izumi, Yoshiyuki Sasaki, Takanori Iwata

**Affiliations:** 1 Department of Periodontology, Graduate School of Medical and Dental Sciences, Tokyo Medical and Dental University (TMDU), 1-5-45 Yushima, Bunkyo-ku, Tokyo, 113-8549 Japan; 2 Department of Nephrology, Faculty of Medicine , Juntendo University, Tokyo, Japan; 3 Department of Internal Medicine, Saiyu Soka Hospital, Saitama, Japan; 4 Division of Periodontology, Department of Oral Interdisciplinary Medicine, Graduate School of Dentistry, Kanagawa Dental University, Kanagawa, Japan; 5 Oral Care Perio Center, Southern Tohoku Research Institute for Neuroscience, Southern Tohoku General Hospital, Fukushima, Japan; 6 Department of Maxillofacial Surgery, Graduate School of Medical and Dental Sciences, Tokyo Medical and Dental University (TMDU), Tokyo, Japan

**Keywords:** Health care, Nephrology, Risk factors

## Abstract

The aim of this study was to investigate the impact of oral hygiene, periodontal diseases, and dental caries on all-cause mortality in hemodialysis. This prospective cohort study included 266 patients with end-stage renal disease who were undergoing hemodialysis. Medical interviews, blood biochemical tests, and comprehensive dental examinations including periodontal pocket examination on all teeth and dental plaque accumulation by debris index-simplified (DI-S), were performed. Survival rates were assessed at a 3-year follow-up. Overall, 207 patients were included in the longitudinal analysis, and 38 subjects died during the follow-up period. Cox proportional hazards analysis of the multivariate model demonstrated that the highest tertile of DI-S had a significantly higher risk of all-cause mortality than the lowest two tertiles after adjustment for age, sex, smoking habit, body mass index, diabetes, prior cardiovascular disease, hemodialysis vintage, high sensitivity C-reactive protein, albumin, and number of remaining teeth (hazard ratio, 3.04; 95% confidence interval, 1.50–6.17; *p* = 0.002). Moreover, the number of decayed teeth significantly increased the hazard ratio to 1.21 (95% confidence interval, 1.06.1.37; *p* = 0.003). This study suggests that accumulated dental plaque and untreated decay, but not periodontal disease, may be independently associated with all-cause mortality in patients undergoing hemodialysis.

## Introduction

Hemodialysis (HD) is initiated in end-stage renal disease. These patients show a high crude mortality rate of more than 20%^1^ due to the high risk of cardiovascular disease (CVD), susceptibility to infection, or immune system degradation. Many factors such as age, presence of diabetes mellitus (DM), history of cardiovascular events, and elevated high-sensitivity C-reactive protein (hsCRP) were reported as risk factors for mortality^2^.

Periodontitis is an inflammatory and infectious disease caused by dental plaque. Periodontitis has demonstrated a high degree of evidence of a relationship with non-communicable diseases such as heart diseases^3,4^, and DM^5,6^ via elevated oxidative stress^7^. The association of periodontitis with chronic kidney disease (CKD) was revealed by an epidemiological investigation^8^ and a recent systematic review and meta-analysis^9^. Several studies have reported that periodontitis may affect the survival rate of patients undergoing HD^10–12^, while a large-scale longitudinal study reported that periodontitis was not associated with an increased risk of early death in adults treated with HD^13^. Whether periodontal disease affects the mortality rate of HD patients remains controversial. Currently available reports are limited, and the findings of these reports are inconsistent between investigations.

It is known that both periodontitis and dental caries are common in CKD patients^14^. Especially in patients undergoing HD, the number of caries and filled teeth is significantly higher than that in healthy individuals^15^. The cause is considered to be a decrease in salivary outflow^16^ and a low frequency of preventative dental care in dialysis patients^17,18^. Impaired masticatory efficiency, oral disease, and subsequent loss of teeth may affect nutrition intake. This may be related to inflammation and malnutrition, which represents a potential modifiable risk factor for cardiovascular disease and mortality.

To more accurately analyze the causal relationship between survival rates and periodontal disease or dental caries, biofilm accumulation on the teeth should be evaluated. It has been reported that oral hygiene behaviors, such as frequency of brushing and use of dental floss, affected the mortality of HD patients^19^. One previous study investigated the impact of oral health parameters, including plaque deposition, on the survival rate of HD patients and reported that they were not significant mortality predictors after adjusting for confounders^20^. However, to our knowledge, careful analysis of the impact of dental parameters on the mortality rate of patients undergoing HD has not yet been performed. Therefore, this study aimed to investigate whether dental markers are associated with mortality in HD patients after adjusting for confounding factors and previously reported risk factors. The design of this study was formulated as follows: 1. participants: hemodialysis patients; 2. exposure factors: high oral plaque accumulation, untreated teeth and periodontal disease; 3. comparison: low oral plaque accumulation, treated caries and periodontal health; 4. outcomes: mortality.

## Results

### Study participants

**Figure 1:**
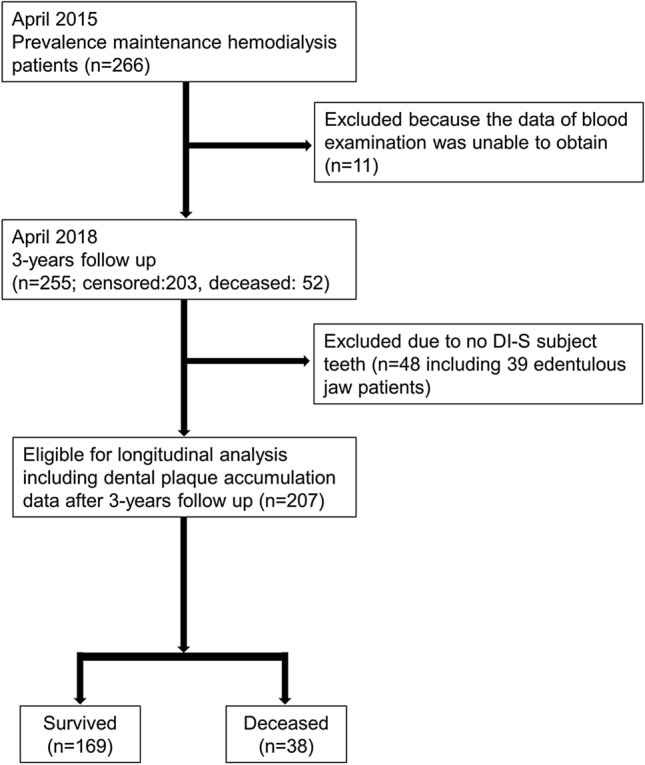
Flow diagram of the subject enrollment process.

A total of 266 patients were assessed for eligibility; eleven enrolled subjects were excluded due to a lack of blood examination data on the day of the dental examination. At the end of the 3-year follow-up, 255 subjects were assessed; 52 subjects had died (20.4%), while others including 11 subjects who were transferred to other hospitals (4 subjects were received kidney transplants) were alive. To assess the impact of dental plaque and periodontal disease, subjects who did not have simplified debris index (DI-S) subjected teeth (n = 48, including 39 with edentulous jaw) were excluded. Finally, 207 patients (38 patients were deceased) were included in the longitudinal analysis (Fig. 1). The baseline characteristics are shown in Table 1. The mean ± standard deviation (SD) age of the study population was 65.9 ± 12.1 years and 135 (65.2%) patients were men. The median dialysis vintage was 64 months. A total of 101 (48.8%) patients had diabetes, and the mean duration of diabetes was 19.0 ± 9.3 years. The mean number of remaining, decayed, and filled teeth was 19.9 ± 7.1, 1.1 ± 2.0, and 8.3 ± 5.3, respectively. The mean DI-S score was 0.99 ± 0.76. The number of patients with healthy, mild, moderate, or severe periodontal disease status was 13 (6.3%), 67 (32.4%), 82 (39.6%), and 45 (21.7%), respectively.

**Table 1:** Baseline characteristics of subjects stratified according to outcome.

Characteristics	All participants (N = 207)	Survivor (N = 169)	Non-survivor (N = 38)	*p* value
Male	135 (65.2%)	111 (65.7%)	24 (63.2%)	0.71
Age (years)	65.9 ± 12.1	64.9 ± 12.5	70.9 ± 9.2	0.01
Smoking				
Non-smoker	84 (40.6%)	70 (41.4%)	14 (36.8%)	0.50
Past-smoker	92 (44.4%)	76 (45.0%)	16 (42.1%)	
Current-smoker	31 (15.0%)	23 (13.6%)	8 (21.1%)	
BMI (kg/m^2^)	21.5 (19.2, 23.5)	21.5 (19.3, 23.6)	19.9 (17.9, 22.9)	0.01
Hemodialysis vintage (months)	64 (33, 115)	62 (33, 116)	74 (35, 107)	0.54
Diabetes	101 (48.8%)	78 (46.1%)	15 (39.5%)	0.15
Duration of diabetes (years)	19.0 ± 9.3	18.8 ± 9.9	20.1 ± 6.7	0.67
Prior CVD event	51 (24.6%)	33 (19.5%)	18 (47.4%)	0.001
Albumin (g/dL)	3.5 (3.3, 3.7)	3.5 (3.3, 3.7)	3.2 (2.9, 3.6)	< 0.001
hsCRP (mg/dL)	0.16 (0.05, 0.45)	0.14 (0.05, 0.34)	0.54 (0.18, 1.84)	< 0.001
**Dental health status**				
Number of remaining teeth	22 (16, 26)	22 (17, 26)	19 (8, 24)	0.006
Number of decayed teeth	0 (0, 1)	0 (0, 1)	1 (0, 3)	0.002
Number of filled teeth	7 (4, 13)	8 (5, 13)	5 (1, 10)	0.006
DI-S	0.8 (0.3, 1.5)	0.8 (0.3, 1.2)	1.5 (0.7, 2.0)	< 0.001
Frequency of tooth brushing (/day)	2 (1, 2)	2 (1, 2)	1 (1, 2)	0.03
Use of interdental cleaning tools	50 (24.1%)	45 (26.6%)	5 (13.2%)	0.03
Periodontal disease				
Healthy	13 (6.3%)	12 (7.1%)	1 (2.63%)	0.54
Mild	67 (32.4%)	54 (32.0%)	13 (34.2%)	
Moderate	82 (39.6%)	70 (41.4%)	12 (31.6%)	
Severe	45 (21.7%)	33 (19.5%)	12 (31.6%)	
PPD≧4 mm (%)	2.9 (0.6, 9.3)	2.8 (0.6, 8.4)	3.8 (0.0, 16.7)	0.42
BOP (%)	7.7 (3.4, 18.7)	7.4 (3.4, 17.3)	11.0 (2.7, 22.3)	0.59
Number of *P.gingivalis* (/ml, log10)	5.3 (4.4, 5.8)	5.3 (4.4, 5.8)	5.6 (5.0, 6.1)	0.17

Values are shown as n (%), mean ± SD, or median (25%, 75%) as appropriate.

BMI, body mass index; BOP, bleeding on probing; BP, blood pressure; CVD, cardiovascular disease; DI-S, simplified debris index; hsCRP, high-sensitivity C-reactive protein; PPD, probing pocket depth.

During the 3-year follow-up period, 38 subjects died (18.4%). Details of the survivors and non-survivors stratified according to outcome are summarized in Table 1. Comparing the two groups, patients in the non-survivor group were older and more likely to have had a history of CVD and a higher serum hsCRP level. Body mass index (BMI) and serum albumin levels were significantly lower in the non-survivor group than in the survivor group. The number of remaining teeth and filled teeth were significantly lower; and the number of decayed teeth was significantly higher in the non-survivor group than in the survivor group. Oral hygiene habits, such as brushing frequency and use of interdental cleaning tools, were significantly worse in the non-survivor group than in the survivor group. The median DI-S was approximately twofold higher in the non-survivor group than in the survivor group (*p* < 0.001).

### Association of dental health status with outcome

**Figure 2:**
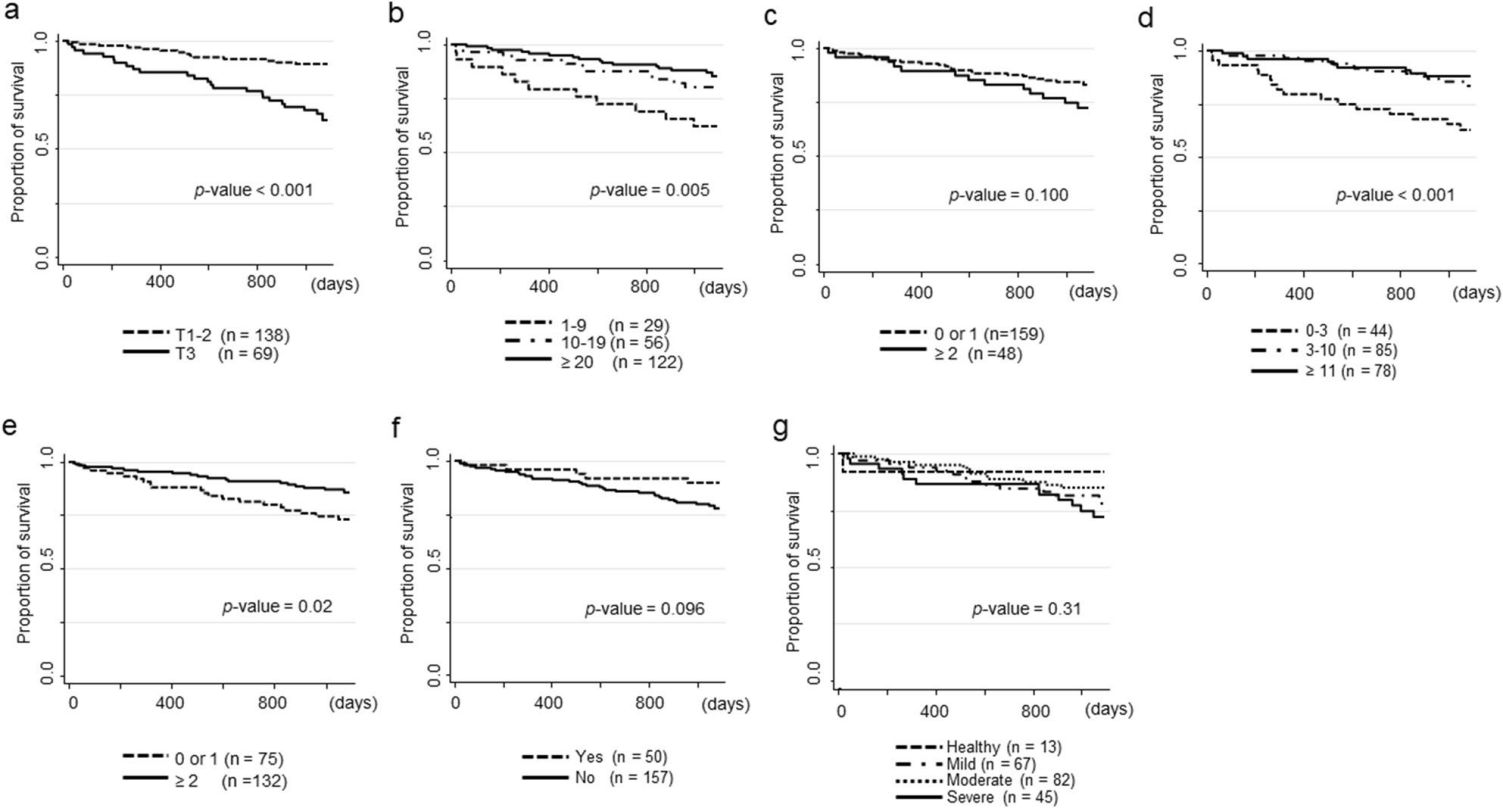
Overall survival for all-cause mortality in hemodialysis patients (n = 207) during the 3-year follow-up period according to dental parameters at baseline; tertiles of DI-S (**a**), categorical variables of the number of remaining teeth (**b**), decayed teeth (**c**), filled teeth (**d**), frequency of tooth brushing per day (**e**), use of interdental cleaning tools (**f**), and classification of periodontal disease (**g**). Significant differences were detected using the log-rank test for equality of survivor function.

To determine the impact of dental markers on the temporal pattern of the occurrence of all-cause mortality, we plotted the overall survival for all-cause mortality according to the follow-up period. For patients in the highest tertile of DI-S, the overall survival for all-cause mortality steeply decreased at a constant rate from the start of the observation (Fig. 2a). In categorical variables of the number of remaining teeth and filled teeth, not decayed teeth, significant differences were detected using the log-rank test for equality of survivor function, although clinical covariates showed an association with risk for all-cause mortality (Fig. 2b, c, and d). Overall survival for all-cause mortality according to the frequency of tooth brushing per day showed significant differences (Fig. 2e), but not the use of interdental cleaning tools (Fig. 2f). In contrast, overall survival for all-cause mortality according to the case classification of periodontal disease did not show significant differences (Fig. 2g).

### Association of DI-S score with all-cause mortality

The subjects were divided into the top and lower tertiles of the DI-S score (T3 group vs. T1-2 group), and their baseline characteristics are summarized in Table 2. The mean age and the ratio of a history of prior CVD events did not differ significantly between the groups. The mean hsCRP value was significantly higher in the T3 group than in the T1-2 group. The mean number of teeth was significantly lower, and the number of decayed teeth was lower in the T3 group compared to the T1-2 group. A higher 3-year mortality rate was observed in T3 compared to T1 or T2.

**Table 2:** Baseline characteristics of subjects and their survival rate at the 3-year follow-up.

	T1-2 (N = 138)	T3 (N = 69)	*p* value
**Baseline characteristics**			
Male	83 (60.1%)	52 (75.4%)	0.03
Age	64.9 ± 12.0	68.2 ± 12.2	0.07
Smoking			
Non-smoker	56 (40.6%)	28 (40.6%)	0.98
Past-smoker	61 (44.2%)	31 (44.9%)	
Current smoker	21 (15.2%)	10 (14.5%)	
BMI (kg/m^2^)	21.4 (19.3, 23.2)	21.5 (191.1, 24.0)	0.87
Hemodialysis vintage (months)	58 (26, 129)	74 (37, 109)	0.50
Diabetes (%)	60 (43.5%)	41 (59.4%)	0.02
Duration of diabetes (years)	18.2 ± 9.3	20.4 ± 9.2	0.25
Prior CVD event (%)	29 (21.0%)	22 (31.9%)	0.06
Albumin (g/dL)	3.5 (3.3, 3.7)	3.5 (3.2, 3.7)	0.20
hsCRP (mg/dL)	0.13 (0.04, 0.40)	0.21 (0.08, 0.92)	0.012
**Baseline dental health status**			
Number of remaining teeth	24 (19, 27)	17 (9, 23)	< 0.001
Number of decayed teeth	0 (0, 4)	0 (0, 2)	0.07
Number of filled teeth	10 (5, 13)	5 (2, 8)	< 0.001
DI-S	0.5 (0.3, 0.8)	1.7 (1.5, 2.0)	< 0.001
Frequency of tooth brushing (/day)	2 (1, 2)	1 (1, 2)	< 0.001
Use of interdental cleaning tools	47 (34.1%)	3 (4.3%)	< 0.001
Dental care within 1 year	84 (60.9%)	28 (40.6%)	0.008
Periodontal disease			
Healthy	12 (8.7%)	1 (1.4%)	0.007
Mild	50 (36.2%)	17 (24.6%)	
Moderate	54 (39.1%)	28 (40.6%)	
Severe	22 (15.9%)	23 (33.3%)	
PPD ≥ 4 mm (%)	2.1 (0.0, 6.0)	6.3 (2.0, 16.7)	< 0.001
BOP (%)	5.8 (2.4, 11.9)	15.6 (6.7, 29.6)	< 0.001
Number of *P.gingivalis* (/ml, log10)	5.3 ± 1.0	5.1 ± 1.0	0.28
**3-year follow up**			
Deceased	14 (10.1%)	24 (34.8%)	< 0.001

The subjects were divided into the top and lower two tertiles of DI-S score (T3 group vs. T1-2 group). Values are shown as n (%), mean ± SD, or med (25%, 75%) as appropriate.

BMI, body mass index; BOP, bleeding on probing; BP, blood pressure; CVD, cardiovascular disease; DI-S, simplified debris index; hsCRP, high-sensitivity C-reactive protein; PPD, probing pocket depth; T, tertile.

The Cox proportional hazards analysis of the univariate model showed that a current smoking habit, history of a prior CVD event, a low albumin level, and a high hsCRP level showed significant HRs associated with the baseline parameters with a 3-year risk of death due to all causes (Table 3). The number of remaining teeth, filled teeth, decayed teeth, DI-S (T3 vs. T1-2), and frequency of daily brushing were significantly associated with the risk of all-cause death in HD patients. Periodontal disease parameters such as the full-mouth ratio of probing pocket depth (PPD) ≥ 4 mm sites, bleeding on probing (BOP)-positive sites, and presence of *P. gingivalis* were not associated (Table 3). The Cox proportional hazards analysis of the multivariate models is shown in Table 4. Model 1 was adjusted for confounding factors such as age, sex, smoking habit, body mass index, the presence of DM. Model 2 was adjusted for above cofounders and number of teeth on the explanatory factors which were assumed to cofound with the number of teeth. Model 3 was adjusted for conventionally-reported risk factors such as prior CVD event, serum hsCRP and albumin level were added in model 1. Multivariate model 3, as well as model 1 and 2, demonstrated that the top tertile of baseline DI-S significantly increased the risk of all-cause death in HD patients after adjustment for age, sex, smoking habit, BMI, DM, serum hsCRP level, albumin, and number of remaining teeth (hazard ratio [HR], 3.04; 95% confidence interval [CI], 1.50.6.17; *p* = 0.002) (Table 4). The number of decayed teeth also significantly increased the HR to 1.21 (95% CI, 1.06.1.37; *p* = 0.003). The proportional hazards assumption was confirmed using graphical diagnostics based on scaled Schoenfeld residuals and log–log survival curves. The test for multivariate model 3 is shown in Supplemental Fig. 1.

**Table 3:** Cox proportional HRs associating baseline parameters with 3-year risk of death due to all causes.

	Univariate model		
	HR	95%CI	p-value
Male	0.90	0.46–1.73	0.75
Age (per years)	1.05	1.01–1.08	0.01
Smoking			
Non-smoker	Ref	–	–
Past-smoker	1.09	0.53–2.24	0.81
Current-smoker	1.68	0.70–4.00	0.24
BMI (kg/m^2^)			
< 18.5	2.21	1.12–4.36	0.02
18.5–24.9	Ref	–	–
≥ 25	0.36	0.09–1.55	0.17
Hemodialysis vintage (per month)	1.00	0.99–1.00	0.51
Diabetes mellitus	1.69	0.88–3.24	0.12
Duration of DM (per year)^a^	1.02	0.97–1.06	0.50
Prior CVD event	3.07	1.62–5.81	0.001
White Blood Cell (per 10^3^/μL)	1.17	1.03–1.34	0.02
Albumin (per g/dL)	0.10	0.04–0.21	< 0.001
hsCRP (per mg/dL)	1.94	1.61–2.35	< 0.001
**Dental status**			
Number of teeth (per tooth)	0.94	0.90–0.98	0.003
Number of decayed teeth (per tooth)	1.18	1.07–1.31	0.002
Number of filled teeth (per tooth)	0.90	0.84–0.97	0.004
**Oral hygiene status**			
DI-S (T3 versus T1-2)	3.78	1.95–7.30	< 0.001
Frequency of tooth brushing (per time/day)			
< 2	Ref	–	–
≥ 2	0.47	0.25–0.90	0.022
Use of interdental cleaning tools	0.46	0.18–1.17	0.10
**Periodontal parameters**			
Severity of periodontitis			
Healthy	Ref	–	–
Mild	2.64	0.33–19.42	0.33
Moderate	1.83	0.24–14.11	0.56
Severe	3.52	0.46–27.10	0.23
PPD ≥ 4 mm (%)			
< 10	Ref	–	–
≥ 10	1.86	0.95–3.64	0.07
BOP (%)			
< 10	Ref	–	–
≥ 10	1.48	0.78–2.80	0.23
Number of *P.gingivalis* (per numbers/ml, log10)	1.30	0.81–2.08	0.28

BMI, body mass index; BOP, bleeding on probing; BP, blood pressure; CVD, cardiovascular disease; DI-S, simplified debris index; hsCRP, high-sensitivity C-reactive protein; PPD, probing pocket depth; Ref, reference.

^a^Records only for subjects with diabetes (n = 101).

^b^Records only for subjects who *P. gingivalis* was detected (n = 115).

**Table 4:** Cox proportional hazard ratios associating baseline dental parameters with a 3-year risk of all-cause mortality: proportional hazards analysis using a multivariate model.

Variable (unit of increase)	Univariate model		Multivariate model 1		Multivariate model 2		Multivariate model 3	
	HR (95% CI)	*p* value	HR (95% CI)	*p* value	HR (95% CI)	*p* value	HR (95% CI)	*p* value
**Dental status**								
Number of teeth (1 tooth)	0.94 (0.90, 0.98)	0.003	0.97 (0.93, 1.02)	0.300	–	–	1.00 (0.95, 1.05)	0.920
Number of decayed teeth (1 tooth)	1.18 (1.07, 1.31)	0.002	1.21 (1.09, 1.35)	< 0.001	1.23 (1.09, 1.37)	< 0.001	1.21 (1.06, 1.37)	0.003
**Oral hygiene status**								
Tooth brushing (twice or more a day)	0.47 (0.25, 0.90)	0.022	0.46 (0.23, 0.96)	0.038	0.48 (0.23, 1.00)	0.049	0.61 (0.28, 1.34)	0.216
Use of interdental cleaning tools	0.46 (0.18, 1.17)	0.104	0.48 (0.19, 1.27)	0.141	0.84 (0.30, 2.34)	0.744	0.84 (0.31, 2.29)	0.734
DI-S (T3 versus T1-2)	3.78 (1.95, 7.30)	0.000	3.40 (1.69, 6.83)	0.001	3.63 (1.70, 7.75)	0.001	3.04 (1.50, 6.17)	0.002
**Periodontal status**								
PPD ≥ 4 mm (10% or more)	1.86 (0.95, 3.64)	0.069	1.70 (0.84, 3.45)	0.141	–	–	1.43 (0.69, 2.94)	0.332
BOP (10% or more)	1.48 (0.78, 2.80)	0.227	1.46 (0.75, 2.82)	0.266	–	–	1.96 (0.97, 3.98)	0.062
Severe periodontitis	1.71 (0.87, 3.40)	0.122	1.33 (0.66, 2.71)	0.430	–	–	1.27 (0.61, 2.65)	0.517

Model 1: Each variable was adjusted for confounding factors: age, sex, current smoking, body mass index, and presence of diabetes.

Model 2: The explanatory factors assumed to coincide with the number of teeth were adjusted for Model 1 + number of teeth.

Model 3: Model 1 + Prior cardiovascular disease event + Albumin + high-sensitivity C-reactive protein.

BOP, bleeding on probing; DI-S, simplified debris index; PPD, probing pocket depth.

Hazard ratios are presented with 95% CI and P value.

## Discussion

To the best of our knowledge, this is the first report to show that dental plaque accumulation and decayed teeth can significantly affect the mortality rate of HD patients, even after adjustment for confounders. We hypothesized that untreated caries, periodontal disease, and poor oral hygiene were exposure factors, and assumed that age, sex, smoking, BMI, number of remaining teeth, and diabetes were confounding factors for the outcome of mortality. Furthermore, untreated caries and DI-S remained significant explanatory variables even after adjusting for previously known risk factors such as a history of CVD, low levels of albumin, and elevated hsCRP levels. Therefore, we considered that there is a significant correlation between both untreated caries and DI-S and the mortality of dialysis patients. However, no rational explanation can be provided from existing evidence regarding the mechanism by which DI-S and untreated caries affect mortality in dialysis patients, except for the pathways involved in a history of CVD or elevated hsCRP levels. Therefore, we consider that DI-S and untreated caries may be markers that influence mortality. However, factors such as access to dental care, health literacy, and socioeconomic factors could not be measured at this time. It is probable that poor oral hygiene, which is caused by insufficient brushing and untreated decay, might be notable aspects of a low level of health consciousness. Indeed, a recent large-scale investigation suggested that improved oral hygiene is positively associated with the decreased occurrence of new-onset diabetes^21^. Oral hygiene behaviors such as frequency of brushing and use of dental floss were reported to affect the mortality of HD patients in a previous report^19^. Although the frequency of brushing and use of interdental cleaning tools were associated with the risk of all-cause death in the univariate hazards analysis in this study, the multivariate model did not demonstrate a statistically significant association.

The plaque control that we focus on in this study is known to be difficult to improve unless continuous strict instructions are provided^22^. Poor plaque control levels may recur even after repeated oral hygiene instruction^23,24^. Therefore, it is assumed that the exposure factor of poor oral hygiene is maintained to some extent during the observation period. In this study, DI-S was used as an index for plaque control. This index is a parameter that can easily evaluate the state of oral cleaning without burdening the patient^25^. It is used as an index for adult brushing instruction, and there is a report that oral hygiene instruction improved the average score from 1.8 to 1.1–1.3^26^. In this study, 1.2 is set as the boundary value, which is considered to be a valid threshold for oral hygiene. The results of this study suggest that remaining dental plaque, not plaque-induced periodontal disease, is a substantial risk factor for mortality. Controversy persists as to whether periodontal disease affects the mortality rate of HD patients^10,11,13^. Poor oral hygiene caused by dental plaque accumulation has been reported to be a risk factor for pneumonia^27,28^, CVD^29^, and endothelial dysfunction^30^. We considered that accumulated dental plaque might cause systemic inflammation that can lead to the occurrence of critical CVD events^31^ or infections through three possible pathways. First, bacteremia caused by daily oral activities such as tooth brushing or chewing^32^ is more likely to occur as the amount of accumulated dental plaque increases^33,34^. In particular, people with poor oral hygiene may be exposed to minor and daily bacteremia, which can cause a systemic inflammatory burden. Periodontal disease contributes to the cumulative long-term inflammatory burden by systemically disseminating inflammatory mediators generated during local inflammatory responses to periodontal pathogens^35^. The second possibility is aspiration pneumonia caused by the aspiration of oral bacteria, as the risk of aspiration pneumonia increases with the amount of dental plaque^36^. A previous prospective cohort study in Japan revealed that periodontal disease is independently associated with pneumonia mortality in hemodialysis patients^11^. The other possibility is dysbiosis caused by the swallowing of oral bacteria. Recently, it has been shown that changes in the structure and function of the intestinal microbiota affect the entire body, such as disorders of the immune system. For example, the repeated swallowing of *P. gingivalis* may cause intestinal dysbiosis^37^. However, the detection of *P. gingivalis* was not a significant predictor of all-cause mortality in the present study. Therefore, further microbiological investigations are required.

The periodontal parameters were not significantly associated with mortality. Periodontal disease is generally an irreversible disease with active and stable phases. Inflammation of the periodontal tissue is more pronounced in the active phase. The case definition of periodontal disease or periodontal pocket depth describes irreversible destruction of periodontal tissue, but does not indicate whether it is active or stable. To compensate for this ambiguity, this study uses BOP as a separate assessment of the inflammatory state of the gingiva. Previous studies have also been controversial with reports of significant or no significant association with periodontitis on the mortality of HD patients. Larger studies are needed to study the influence of periodontal inflammation in patients with HD.

The limitations of this study were that it included the findings of a single dental examination measured during the follow-up period. In this study, the examination value at baseline was used as an explanatory variable. Although, the analysis assumes that these exposure factors continue to be equivalent during the observation period. Therefore, careful interpretation of the presented conclusion is required because of the substantial methodological weaknesses. The residual confounding may be a factor due to insufficient sample size and a broad range of clinical factors. As the study includes data from a single-center and an all-Japanese population, the presented analytical model is not applicable to other populations such as different regions or races. In addition, there may be unknown confounding factors, such as socioeconomic status and accessibility to dental care. A well-designed prospective intervention study is needed to validate our findings, which suggest that improved dental hygiene may prolong the life of HD patients.

In conclusion, the present findings suggest that dental plaque accumulation and untreated decay are independently associated with all-cause mortality in HD patients. Because plaque accumulation levels can be improved with behavior modification by the patients themselves and the dental professions’ support, the present findings on DI-S may have certain clinical implications for the dental care of patients undergoing maintenance hemodialysis.

## Materials and methods

### Study participants

This study enrolled 266 patients with end-stage renal disease undergoing HD at an outpatient hemodialysis clinic located in the Tokyo metropolitan area in April 2015. All participants provided informed consent. The primary outcome measure was all-cause mortality from the time of baseline examination in the study. Participant survival rate was assessed based on medical records at a 3-year follow-up in April 2018. This study was approved by the Research Ethics Committee of Tokyo Medical and Dental University (D2014-126) and complied with the Declaration of Helsinki. This trial followed the CONSORT guidelines and is registered in the University Hospital Medical Information Network (UMIN) Clinical Trials Registry (UMIN000039845).

### Clinical examination and laboratory measurements

We performed a medical interview, blood biochemical test including hsCRP level, blood pressure measurement, and physical measurements. A thorough medical history was taken at the time of the study enrollment by a trained physician. Prior CVD was defined as a medical history and clinical findings of coronary artery, cerebrovascular, and peripheral vascular diseases. The details have been described in our previous study^38^.

A comprehensive dental examination was performed. The number of remaining teeth was recorded as it is not only an important factor in oral function but can also be a cumulative consequence of the progression of periodontal disease. To evaluate dental health, the number of decayed, missing, and filled teeth were recorded following the oral health assessment criteria by WHO as the DMFT index^39^. Oral hygiene status was evaluated according to the DI-S^40^. Briefly, the six surfaces examined for the DI-S were selected from four posterior and two anterior teeth, known as the Ramfjord index teeth^41^, for periodontal screening. In the posterior dentition, the buccal surfaces of the upper first molars and the lingual surfaces of the first molar were screened, and at times the second molar was screened. In the anterior teeth, the labial surfaces of the upper right and the lower left central incisors were scored. In the absence of either of these anterior teeth, the central incisor on the opposite side of the midline was substituted. The criteria for classifying debris were 0, no debris; 1. debris covering not more than one-third of the tooth surface; 2. debris covering more than one-third, but not more than two-thirds; and 3. debris covering more than two-thirds of the exposed tooth surface. The average score of six possible surfaces was calculated. Periodontal examination of tooth mobility, PPD, clinical attachment level, and BOP at 6 sites on all residual teeth was performed with a manual probe (PCP‐UNC 15, Hu-Friedy, Chicago, IL) by experienced periodontists. Based on the periodontal examination findings, the periodontal case classification was diagnosed at the time of examination according to the Centers of Disease Control and Prevention / American Academy of Periodontology (CDC/AAP) case definition^42^. Resting saliva was collected into a designated tray for 1 min to measure the number of *P. gingivalis* using real-time polymerase chain reaction (PCR)^43,44^. Samples were kept in a freezer at –80 °C until used for the extraction of bacterial DNA (QIAmp DNA Mini Kit; Quiagen, Valencia, CA, USA). The real-time PCR was performed using a PCR reaction mixture (Premix ExTaq, Takara-bio, Shiga, Japan) with the one cycle at 95 °C for 30 s followed by 40 cycles at 95 °C each for 5 s, and at 60 °C for 30 s (Thermal Cycler Dice Real Time System II, Takara-bio, Shiga, Japan). The primers and probe were below; Forward: 5′-TAGCTTGCTAAGGTCGATGG-3′, Reverse: 5′-CAAGTGTATGCGGTTTTAGT-3′, TaqMan Probe: FAM-TGCGTAACGCGTATGCAACTTGCC-TAMRA.

### Statistical analysis

All variables are expressed as numbers (percentages) for categorical data and as means ± SD or median and interquartile ranges for continuous data with and without a normal distribution, respectively. For analytical purposes, patients were stratified according to survivors and non-survivors, or their tertiles of DI-S levels. Differences between groups were assessed using t-test, Kruskal–Wallis test, Mann–Whitney test, or Fisher’s exact test, depending on the distribution (normal or skewed, respectively). Kaplan–Meier survival estimates were calculated based on dental markers. Significant differences were detected using the log-rank test for equality of survivor function. Univariate Cox proportional hazards analysis was used to examine the association of baseline variables with all-cause mortality. Multivariate-adjusted HR values were calculated after adjustment for confounders such as age, sex, smoking habit, body mass index (BMI), diabetes, prior CVD, HD vintage, hsCRP and albumin levels. The variables were applied based on the previous studies of multivariate models of all-cause mortality in HD patients^2^. Because the associations of BMI or DI-S with outcomes were not linear, these two explanatory variables were subjected to the Cox proportional hazard model as categorical variables. The proportional hazard assumption in the Cox models was tested. All *p* values were 2-sided, and those < 0.05 were considered statistically significant. Statistical analysis was performed using a statistical software (STATA version 15.0; Stata, College Station, Texas, USA).

## Supplementary information

Supplementary Information 1.

Supplementary Information 2.
