# Supplementary figures and images for: Poor oral hygiene and dental caries predict high mortality rate in hemodialysis: a 3-year cohort study
Source: Sci Rep. 2020 Dec 14;10:21872. doi: 10.1038/s41598-020-78724-1 (PMC7736314; doi:10.1038/s41598-020-78724-1)

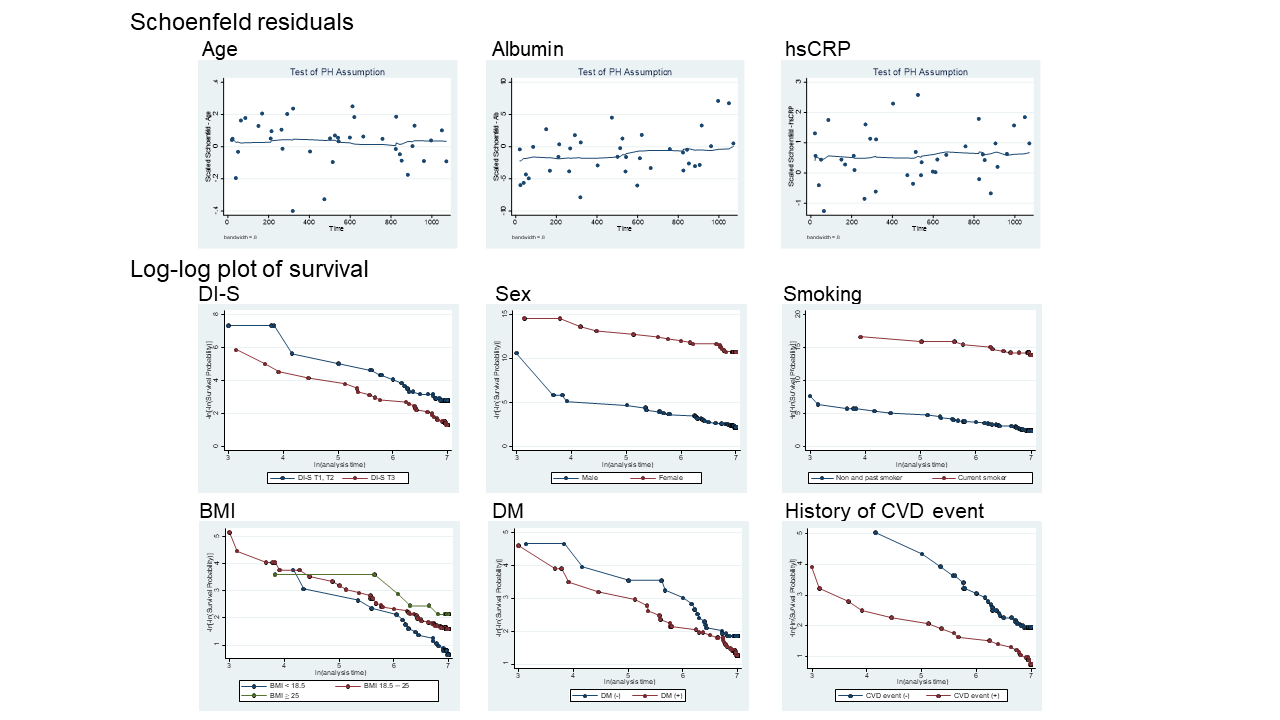

Supplement: Supplementary file 1 — Supplementary Information 1. [file 41598_2020_78724_MOESM1_ESM.tif]
