# Supplementary material for: Poor oral hygiene and dental caries predict high mortality rate in hemodialysis: a 3-year cohort study
Source: Sci Rep. 2020 Dec 14;10:21872. doi: 10.1038/s41598-020-78724-1 (PMC7736314; doi:10.1038/s41598-020-78724-1)
Supplement: Supplementary file 2 — Supplementary Information 2. [file 41598_2020_78724_MOESM2_ESM.docx]

**Poor oral hygiene and dental caries predict high mortality rate in hemodialysis: A 3-year cohort study**

Koji Mizutani, DDS, PhD*^1#^, Risako Mikami, DDS, PhD^1#^, Tomohito Gohda, MD, PhD^2^, Hiromichi Gotoh, MD, PhD^3^, Norio Aoyama, DDS, PhD^4^, Takanori Matsuura, DDS, PhD^1^, Daisuke Kido, DDS, PhD^1^, Kohei Takeda, DDS, PhD^1^, Yuichi Izumi, DDS, PhD^1,5^, Yoshiyuki Sasaki, DDS, PhD^6^, and Takanori Iwata, DDS, PhD^1^

**Supplemental Figure 1.** The proportional hazards assumption test for multivariate model 3. The proportional hazards assumption was confirmed using graphical diagnostics based on scaled Schoenfeld residuals and log-log survival curves.
